# Melanoma Epidemiology and Early Detection in Europe: Diversity and Disparities

**DOI:** 10.5826/dpc.1003a33

**Published:** 2020-06-29

**Authors:** Ana-Maria Forsea

**Affiliations:** 1 Oncologic Dermatology Department, Elias University Hospital; Carol Davila University of Medicine and Pharmacy, Bucharest, Romania

**Keywords:** melanoma, epidemiology, early detection, disparities, Europe

## Abstract

Melanoma claims annually more than 20,000 lives in Europe and is an important public health burden through its continuously increasing incidence and with its high mortality, costs, and complexity of care in advanced stages. Epidemiological surveillance is indispensable for the research into its causes, new prognostic markers, and innovative therapies, as well as for the building of efficient cancer control plans. However, important differences in the sources and availability of accurate epidemiological data exist among European countries and regions, contributing to a heterogeneous picture with 20-fold differences in the reported national melanoma incidence rates, divergent mortality trends, and solid disparities in survival across the Continent. Countries in the eastern half of Europe report the lowest incidence rates, but high case fatality, persisting and increasing mortality, a higher proportion of thicker tumors and late diagnosis, and lower survival rates. They are the least well equipped with quality cancer registration and reporting, and they lag behind in efficient cancer control plans implementation. This review highlights the main differences in melanoma epidemiology across Europe, together with an insight into their underlying causes in the areas of melanoma registration, early diagnosis, and prevention. These differences should be acknowledged and understood by physicians, researchers, and all stakeholders involved in improving melanoma care and outcomes, as no one-size-fits-all solution can tackle the melanoma problem in Europe. Instead, there is a need for nuanced strategies, adapted to the heterogeneous national and regional contexts, that would build on European diversity to eliminate the outcome disparities.

## Introduction

Melanoma is a tumor with high impact through its rapidly growing incidence, high mortality, and increased complexity and costs of care in advanced stages. Research efforts are fervently unfolding worldwide to shift its diagnosis toward earlier stages, to prevent its occurrence, and to develop breakthrough treatments. Avid observation of the epidemiological data and trends is ongoing, to capture the first success signs of these initiatives through the changes in melanoma incidence or mortality, and to scour for clues for new risk factors or prognostic markers. Epidemiological data are explosively accumulating through the continuous improvement and expansion of cancer registries. The digital age offers unprecedented facility of data collecting, sharing, and comparing across countries, regions, and centers. The picture of melanoma burden in Europe becomes thus ever sharper, and so its heterogeneity becomes more apparent. The most visible, also to the public, are the glaring outcome disparities. Melanoma survival exceeded 90% for 5-year relative rate for Nordic or Western countries, but is below 60% in Eastern Europe for people diagnosed as recently as this decade [1]. This is but the tip of the iceberg, whose base reaches deep into the disparities across the whole spectrum of melanoma care, from prevention and early diagnosis to access to treatment, and—not least important—to the availability and accuracy of the epidemiological data.

This review aims to highlight the main differences in melanoma epidemiology across Europe, bringing insight into their underlying causes in the areas of melanoma registration, early diagnosis, and prevention. It aims to offer a differentiated depiction of the challenges of melanoma epidemiological surveillance, in support of the concept that in order to tackle this tumor, no one-size-fits-all solution can work, but nuanced actions, adapted to the heterogeneous national and regional context in Europe, are necessary.

## Sources of Epidemiological Data for Melanoma in Europe

Epidemiological analyses, however complex, are only as good as their data sources. The foundation of cancer epidemiological surveillance consists of the population-based cancer registries (PCRs). PCRs are complex organizations destined to systematically collect, store, analyze, and report the data about all cancer cases occurring in a certain population, usually of a specific geographical area [2,3]. They are the fundamental source of objective information on cancer cases, but their function and performance is conditioned by the organization of the health systems in which they operate and by their legal, economic, social, and cultural context at the national or regional level. These are highly heterogeneous in Europe, and so are PCRs’ quality, function, and output [4]. These differences have far-reaching consequences, as accurate and complete PCR data are indispensable for all aspects of epidemiological research and cancer control planning [2,4–7].

Cutaneous melanoma is reported mostly to general PCRs, and these are so far the mainstay source of epidemiological data on melanoma in Europe. Close to 200 national or regional PCRs cover together approximately 60% of the population of Europe, with increasing trend [8,9]*.* Their coverage rate, data output, and quality differ largely, with the countries in South-Eastern and Eastern Europe (SEE) lagging constantly behind [4,8,10–14].

Twenty-two European countries have quality national PCRs, covering the entire population [4,8, 15, 16]*.* In contrast, 9 countries in SEE report only estimated incidence rates, calculated from partial registration data and neighboring countries’ quality registries [4,16,17].

The vast majority of PCRs report incidence and mortality data, but only 60% of them reportedly collect follow-up data for estimating cancer survival rates [8]. The SEE region has the lowest percentage of registries reporting on survival rates [8], while it estimates the lowest survival rates in Europe, including for melanoma [4,18]. The SEE region has also the lowest proportion of PCRs producing more advanced data on stage at diagnosis, details and delay of first treatment, and compliance with treatment guidelines, while Nordic PCRs score consistently at best across all these parameters [8,10].

Reporting and publishing of European PCR data are also highly variable [4,5, 8, 14], and SEE countries are the least represented in general cancer and melanoma-focused studies [4,19–21]. Centralized data at the European level, with an endeavor for standardization, were made available in the last decades through several platforms including the European Network of Cancer Registries (ENCR) and the International Agency for Research on Cancer (IARC) portals [9,22], as well as through high-resolution pan-European studies [4,22]. A major step forward in ensuring harmonized, centralized, and up-to-date reporting of European cancer data was the launching in 2018 of the European Cancer Information System (ECIS) [17]. Grounded in joint efforts of ENCR-Joint Research Centre and IARC, the ECIS reports national incidence and mortality estimates for the current year, historical recorded incidence and mortality indicators as available, and estimated 5-year survival rates for European Union (EU) and European Free Trade Association (EFTA) countries [21].

Melanoma registration through PCRs faces additional specific challenges. While most PCRs reportedly collect data from pathology reports, hospital records, and death certificates [5], significantly fewer collect data from public or private outpatient clinics, hospices, or general practitioner records [9,10], where many localized melanomas are diagnosed and initially excised. Different specialties involved in early melanoma diagnosis and treatment, from dermatologists to general practitioners and surgeons, have different patterns of cancer case reporting [4]. While stage at diagnosis is collected by most PCRs, fewer than 40% of them use the tumor-node-metastasis (TNM) classification for this purpose [15], and important parameters such as melanoma thickness or genetic profiling are often not recorded [23]. These concur with underreporting of melanoma cases and limitations in the analysis of cutaneous melanoma prognosis, early diagnosis, and care quality across Europe.

## Melanoma Incidence and Mortality in Europe

With the caveat of the variability of PCR output, as exposed above, the data available draw a highly heterogeneous epidemiological picture of melanoma in Europe. We signaled 19-fold differences in the estimated incidence rates across the Continent [24], with a sharp north-to-south, west-to-east gradient. Ten years later, the current ECIS estimates maintain this trend, with the highest incidence rates in Norway (29.6 age-standardized rate, world population [ASWR]) and the lowest in Romania (3.4 ASWR), with 13.5 (ASWR) European average [17]. Looking closer, 3- to 4-fold differences exist between neighboring countries’ rates, such as Romania and Hungary or Poland and Germany. As the population genotypic and phenotypic characteristics, sun exposure behavior, and UV index do not diverge much between these countries sharing borders, it is likely that these incidence disparities reflect inequalities in melanoma case diagnosis and registration, beyond the true burden of new melanoma.

Estimates of mortality rates vary less between countries, from the lowest of 1.1 ASWR in Malta, to 3.5 ASWR in Norway, with an average of 1.7 for the EU + EFTA countries [17]. Norway is an outlier, at a distance from the next mortality rates in the neighboring Nordic countries (ranging 1.7–2.7 ASWR) [17]. Comparable mortality rates between Central and Eastern European (CEE) and Western European countries, but with significantly lower incidence rates reported in CEE countries, point to much higher case-fatality rates in Eastern European countries [25].

Mortality rates are uniformly higher in men than in women, ranging across Europe from 0.9 to 2.8 in women, and from 1.3 to 4.2 ASWR in men [17]. The Nordic countries and Switzerland show the highest gender difference in mortality rates, with the maximum in Finland (1.1 in women vs 2.9 in men). Melanoma continues to claim more than 20,000 lives annually in Europe [17,24], with the highest proportion in CEE countries [24].

## Melanoma Survival in Europe

Melanoma survival in Europe has been constantly rising [26], but this encouraging news is far from uniform across the Continent. The last EUROCARE-5 analysis of recorded data from 99 European PCRs shows that the 5-year relative survival rate for melanoma patients diagnosed between 2002 and 2007 is 86.58% for Europe, with the highest rates reported in Nordic countries (90%) and the lowest in Eastern Europe (69.78%) [26]. At the individual country level these rates go even lower, for example, in Bulgaria to 40.73% and in Poland to 55.35%. Because of data missing from registries, not all European countries have survival data estimated by EUROCARE/ECIS. In an attempt to fill this gap, our earlier study used the incidence-mortality ratio as proxy for estimating survival for all 40 European countries for which incidence and mortality data were available in IARC/Globocan [25]. Our estimation paralleled the hard data of EUROCARE, as the incidence-mortality ratio increased sharply from east and south to north and west, ranging from 1.86 and 2 for Albania and Romania, to 8.35 in Switzerland.

As these dramatically low rates in Eastern Europe, both estimated and recorded, stem from the era before the widespread introduction of innovative therapies for advanced melanoma, they likely reflect disparities in early diagnosis and management of primary tumor across the Continent. This is underlined by recent survival analyses stratified by tumor stage and thickness, showing that while the 5-year relative survival rate increased significantly from 2000 to 2013, this increase was no longer seen after adjustment for T stage [27]. Increased survival appears thus due mostly to the increased earlier diagnosis of thinner tumors, with good prognosis. This finding emphasizes the urgent need to step up efforts in this direction in the Eastern European countries.

## Melanoma Epidemiology Trends in Europe

Melanoma incidence has been constantly increasing in all European regions for the last few decades. During the 1995-2012 period, a statistically significant increase in the incidence of invasive melanoma was reported, with an average annual percent change (AAPC) of 4.0% in men and 3.0% in women [20]. The increase tended to be higher in Southern and Eastern Europe than in Nordic countries [19,20]. In SEE countries, incidence rates increased over the period 2001–2010 across all age groups (25–49 years old, 50–69 years, and 70+ years) [28], although the steepest increases were noted in the older groups. Incidence increased more rapidly in women than in men.

In contrast, in Nordic countries, a similar PCR-based analysis over the period 1990–2010 [19] showed a stabilization of incidence rates and even a decrease (in Norway) in the youngest groups, with a marked increase persisting in over 70-year-olds. Incidence increased more rapidly in women than in men under 70 years old, but the trend was reversed after this age.

The marked increase in incidence noted in SEE countries is likely related at least partially to increased diagnosis and case registration in recent years, and as these improvements continue, the reported epidemiological data will come closer to reflecting the real melanoma burden in these countries.

Mortality has increased in Europe, although at slower rates than incidence. In the period 1995–2013 mortality rates continued to increase in Northern (AAPC 1.6%–5.4%) and Western Europe (AAPC 1.0%–2.1%) [20], while some countries (Austria, Switzerland) reported a flattening or even a slight decrease in overall mortality [20]. Mortality trends were generally more favorable for women than men, and more favorable—even decreasing—in younger age groups, while elder men bore the highest burden of mortality increase [20,29]. In South-Eastern and Eastern Europe the trends are more divergent. In the youngest age groups (25–49 years old) mortality nonsignificantly declined in most countries in the period 2000–2010 [28]. For the middle age group (50–69 years old), country reports are heterogeneous, with decreasing trends in a few countries such as Czech Republic and Slovakia but increasing in the others, with a maximum for men in Serbia (AAPC 3.6%) and for women in Slovenia (AAPC 6.0%). The oldest groups show uniformly a marked mortality increase in both genders, up to 8% AAPC in Serbia. These highly heterogeneous data in close neighbors suggest differences in reporting besides disparities in melanoma diagnosis and treatment.

The burden of melanoma is expected to continue to rise in the future across Europe. The number of reported new cases is expected to rise as the population ages and as awareness and early detection increases, while sun-related behaviors do not seem to change significantly. The incidence rates are projected to rise until 2026 in Northern Europe and possibly stabilize afterward [30]. In South-Eastern and Eastern Europe, the incidence rates are likely to continue to increase significantly also as a result of improving diagnosis, registration, and reporting [4,25].

It is difficult to speculate on future developments in mortality, as since the 2012–2015 innovative, efficient therapies have been introduced in European countries, yet access to them is highly uneven across the Continent [31].

## Melanoma Thickness in Europe

Reported melanoma thickness has decreased markedly over the last decades. In Germany the median Breslow thickness decreased from 1.81 mm in 1980 to 0.53 mm in 2000 [32]. In Switzerland the incidence rates of thin melanoma (<1 mm) increased approximately 4-fold between 1980 and 2010, while the incidence rates of thicker tumors remained relatively stable [33]. Nordic and Western countries report similar trends, leading to 60%–70% of melanomas being diagnosed under 1 mm in 2010–2015 [27,34–37]. A recent PCR-based study [20] found that since 1995 the incidence rates increased markedly both for in situ (AAPC 7.7% in men; 6.2% in women) and thin melanoma (AAPC 8.3% in women; 10% in men) in 12 countries situated, with one exception, in the Western part of the Continent. In contrast, thicker melanoma (>1 mm) incidence rates also increased, but much slower (APC 3.3%-2.2%) [20].

In the eastern part of Europe the picture is different, as few reports on melanoma thickness exist, mostly from individual centers, whereas PCRs collect thickness data unevenly [23]. In Serbia between 2005 and 2010 the median Breslow thickness reported was 3 mm [38]. In Estonia the proportion of thin melanoma (T1 of TNM classification) increased from 7% in men and 14% in women in 1992 to 34% in men and 41% in women in 2010–2012, while the proportion of T4 tumors decreased slightly [39]. In Croatia the mean Breslow thickness reported in 2012 was 2 mm, with 75% of T4 tumors found in men and 60% of T1 tumors in women [40]. A regional reference center in Romania reported a stage distribution of 18% T1 and 48.73% T4 [41]. An expert survey in 33 European countries estimated that melanomas thicker than 2 mm represented 46% of melanomas diagnosed in Eastern Europe, vs only 19% in Western Europe [42]. Encouragingly, earlier diagnosis of thinner tumors appears also in the CEE countries with longer-established PCRs, such as the Czech Republic, where between 1977 and 2008 the incidence rates increased at an estimated annual percentage change of 38%, for T1 tumors vs 5% for T4 tumors [43].

All available data suggest a general trend, albeit at different speeds, of a stronger increase in incidence of in situ and thin tumors, while thicker tumors continue to increase in incidence although at a significantly slower rate [29,33,35–37,44]. This persistent proportion of thick tumors detected may explain the persisting and even increasing mortality rates across the Continent, and it points to the necessity to intensify the efforts of primary and secondary prevention.

## Melanoma Prevention Campaigns in Europe

The disparities in incidence and early diagnosis across the Continent relate to significant differences in the level of population awareness and available primary and secondary prevention campaigns. Nordic and Western European countries have a several decades long tradition of public education campaigns for skin cancer prevention and early detection [34,37,42,45,46]. Germany is the only country implementing a national skin cancer screening program [47–49], while diverse, high-risk-based selective screening programs are reported in Western countries [50–52]. Skin self-examination is promoted, pigment lesion clinics that fast-track patients with suspected melanoma [53–55] succeed in increased detection of thinner tumors, and various digital tools promoting public awareness, skin self-examination, or skin cancer risk estimation are developed [56–58].

This is in sharp contrast with the paucity of interventions reported in the eastern half of the Continent. In an expert survey conducted in 32 European countries, fewer respondents in Eastern Europe compared with Western Europe reported the presence of governmental (12% vs 46%) or nongovernmental (35% vs 65%) initiatives for skin cancer prevention [42]. Euromelanoma, the pan-European skin cancer prevention campaign [45,59,60], has achieved major progress for most countries in this region, where it was the first initiative of skin cancer prevention at a national scale [45]. It was also the first nationwide public skin cancer screening campaign ever organized in all CEE and Baltic states [45]. In CEE countries, potential screening programs are also hampered by obstacles including low population awareness, insufficient and geographically maldistributed diagnostic capacity and health workforce, but also insufficient epidemiological quality control, political support, and funding [61,62].

Accordingly, the level of public awareness for melanoma prevention and early detection maintains the west-east divide. In France, for example, 92% of the surveyed general population knew about sun risks and sun protection, 60% knew the ABCDE rule, and 43% of respondents had consulted a doctor at least once for a suspect skin lesion [63]. In contrast, in Romania, 90.76% of all surveyed skin cancer patients were not warned by any doctor about their skin cancer risk and more than 65% of them never had a medical skin check before diagnosis [64].

## Melanoma in Europe: Further Disparities

Beyond discrepancies in prevention, early detection, and registration, the significant differences in melanoma burden and outcome across Europe are rooted in disparities along the whole continuum of melanoma care, with CEE countries lagging behind. Total health expenditure per capita correlates closely with estimated melanoma survival in Europe [25], and this parameter is still very heterogeneous, ranging in 2016 from 5,600 euros in Luxembourg to 400 euros in Romania, with Nordic countries at the top end and Eastern European countries at the bottom [65]. The budget available to PCRs per new cancer case averaged 150 euros in the northwest region and only 34 euros in Eastern Europe [66]. The access to essential early diagnostic facilities such as dermoscopy and digital dermoscopy is uneven [67,68]. Furthermore, access to new, innovative, highly efficient treatments is limited or delayed in the eastern half of the Continent [31,69], as is access to multidisciplinary care [70]. On a deeper level, disparities in the process quality, availability of resources, and governance of health care systems that ground the differences in cancer care outcomes [71] are at the basis of the “iceberg” of melanoma burden and prognosis inequalities.

## Conclusions

The burden of melanoma in Europe is unevenly distributed among countries and regions. Important disparities exist across the Continent in the collection and availability of accurate epidemiological data, in the capacity of early diagnosis, and in the prevalence of preventive efforts, reflecting into large differences in incidence, mortality, and survival figures and trends. These differences must be mapped and understood, in order to devise adapted, efficient strategies of melanoma prevention and care that would build on the European diversity to eliminate the outcome disparities.
